# Model variations for tracking the trunk during sports testing in a motion capture lab

**DOI:** 10.3389/fspor.2024.1429822

**Published:** 2024-07-19

**Authors:** Sophia Ulman, Alex Loewen, Ashley Erdman, Sylvia Õunpuu, Ross Chafetz, Kirsten Tulchin-Francis, Tishya A. L. Wren

**Affiliations:** ^1^ Orthopedic and Sports Medicine Center, Scottish Rite for Children, Dallas, TX, United States; ^2^ Department of Orthopedic Surgery, University of Texas Southwestern Medical Center, Dallas, TX, United States; ^3^ Center for Motion Analysis, Connecticut Children’s Medical Center, Hartford, CT, United States; ^4^ Motion Analysis Center, Shriners Hospital for Children, Philadelphia, PA, United States; ^5^ Department of Orthopedic Surgery, Nationwide Children’s Hospital, Columbus, OH, United States; ^6^ Jackie and Gene Autry Orthopedic Center, Children’s Hospital of Los Angeles, Los Angeles, CA, United States

**Keywords:** biomechanics, trunk, knee, return-to-sport, motion analysis

## Abstract

**Introduction:**

As motion capture technology becomes more popular for athlete monitoring and return-to-play evaluation, it is imperative that trunk mechanics are modeled similarly across participants. The purpose of this study was to determine how adjusting marker placement at the sternum or removing potentially occluded markers for purposes of tracking the trunk segment influences trunk kinematics during gait and a drop vertical jump (DVJ).

**Methods:**

Sagittal plane trunk angles of 18 participants were computed for a Definition Model and three trunk model variations. Model variations were specifically chosen to avoid difficulties with placement of the sternum and/or thorax markers in female participants due to sports bra coverage and/or occlusion. Intraclass correlation coefficients were computed per trunk model variation to determine agreement with the Definition Model.

**Results:**

The Mid-Sternum model, in which the xiphoid process marker was adjusted to the midpoint of the xiphoid process and jugular notch, exhibited the least discrepancies and excellent agreement with the Definition Model across both tasks. Alternatively, the No-Thorax model, in which the thorax marker was removed, exhibited the greatest kinematic differences during the DVJ and moderate to excellent agreement across both tasks.

**Conclusion:**

The marker set chosen to track trunk motion during dynamic tasks must include locations that can be placed similarly on all participants. Based on these findings, the xiphoid process marker may be adjusted superiorly prior to the collection of dynamic trials. The recommended model for tracking the trunk segment includes marker placements on the jugular notch, mid-sternum, and 1st and 10th thoracic spinous processes.

## Introduction

Three-dimensional (3D) trunk position during high-impact, sport-specific tasks has been reported to be a strong indicator for injury risk in the athlete population (1–3) and is important to consider in return-to-play (RTP) decision-making (4, 5). This is primarily because significant displacement and/or poor control of the trunk throughout a dynamic movement influences the knee joint's ability to maintain an optimal, inline position (3, 6, 7). Furthermore, recent literature has indicated that targeted training of trunk dynamic control may improve high-risk knee positions and loading patterns associated with anterior cruciate ligament injury (7). Therefore, it is imperative that functional assessments employed to gauge recovery or RTP readiness allow for objective and reliable evaluation of trunk position (7).

Traditionally, visual (2D video) assessments of movement have included scoring items specific to trunk control that are binary or scored on a Likert scale (8, 9). More recently, as technology becomes more integrated into the clinic, wearables have also become popular to compute a variety of trunk motion measures, such as trunk angles or velocities (10–12). The most recent technological advancement in motion analysis is the release of markerless motion capture, which employs artificial intelligence to model movement and does not require the extensive preparatory time needed to instrument a patient with motion capture markers. However, recent reports indicate a lack of agreement with trusted marker-based systems, specifically regarding trunk flexion during dynamic, sport-like tasks (13–15). Until the validity and reliability of markerless motion capture improves and clinical efficacy is established, traditional marker-based motion capture will continue to be the norm. Furthermore, as motion capture technology becomes more popular for athlete monitoring and RTP evaluation (16–18), it is critical that movement patterns are modeled similarly across patients, especially during dynamic tasks (19). A systematic review by Negrini et al. concluded that clinical literature focusing on trunk motion analysis is severely lacking. As such, standardization for modeling the trunk during dynamic movement is critical (20).

In previous work, marker set variations for defining the trunk segment were evaluated and analyses concluded that relatively small adjustments to marker placement, especially at the thoracic and sternum locations, altered kinematic outcomes during gait and a drop vertical jump (DVJ) task (21). Based on these findings, a single marker set was ultimately recommended with specific options for adjustments as needed. The marker set recommended for defining the trunk segment during the static (standing) trial included the 1st thoracic spinous process (T1), 10th thoracic spinous process (T10), the deepest point of incisura jugularis or suprasternal notch (CLAV), and the xiphoid process (XP). Additionally, adjustments that were shown to not significantly alter kinematic outcomes were described for instances with female athletes when sports bra coverage was occluding the T10 or XP placements.

However, while these adjustments may be sufficient in some cases, they are not a universal solution. Several studies have investigated alternative trunk marker sets based on specific study aims and/or visibility during testing. For example, Orantes-Gonzalez et al. reported moderate to excellent reliability of an anterior-only marker set, designed to model trunk movement during load carriage tasks, compared to a variety of alternative marker sets including the International Society of Biomechanics (ISB) recommended model (7th cervical spinous process, CLAV, XP, and 8th thoracic spinous process) (22). Alternatively, for treadmill running, Ekizos et al. reported that trunk kinematics computed using variations of a posterior-only marker set were significantly different based on placement of a single spine marker, specifically between placement of the 2nd lumbar spinous process and more proximal marker locations (23). Another option for modeling the trunk reliably and consistently across patients may involve altering the marker set only in the dynamic trials for tracking purposes after the trunk segment has been defined in the static trial with the recommended model.

In assessing movement quality, accurate and reliable data is essential for decision-making. As ACL injury rates increase among athletes (24), practitioners are becoming more cautious in returning athletes back to competition if movement deficiencies are observed (25, 26), such as a lack of trunk control. Given athletes present in clinic with a variety of body types and sizes, it is important that objective movement assessments are consistently reliable across patients, and data is not skewed by human or technology-driven error. Therefore, the purpose of this study was to determine whether variations in tracking the trunk segment, specifically by adjusting marker placement at the sternum or removing potentially occluded markers, influenced trunk kinematics during gait and a DVJ task. It was hypothesized that adjusting a trunk marker would influence trunk kinematics less than removing a marker, resulting in a three-marker tracking model. Additionally, among the three-marker models, it was hypothesized that the model lacking the XP marker would influence trunk kinematics the least. Influence on trunk kinematics will be tested by comparing each alternate tracking model to the definition model.

## Methods

**Figure 1:**
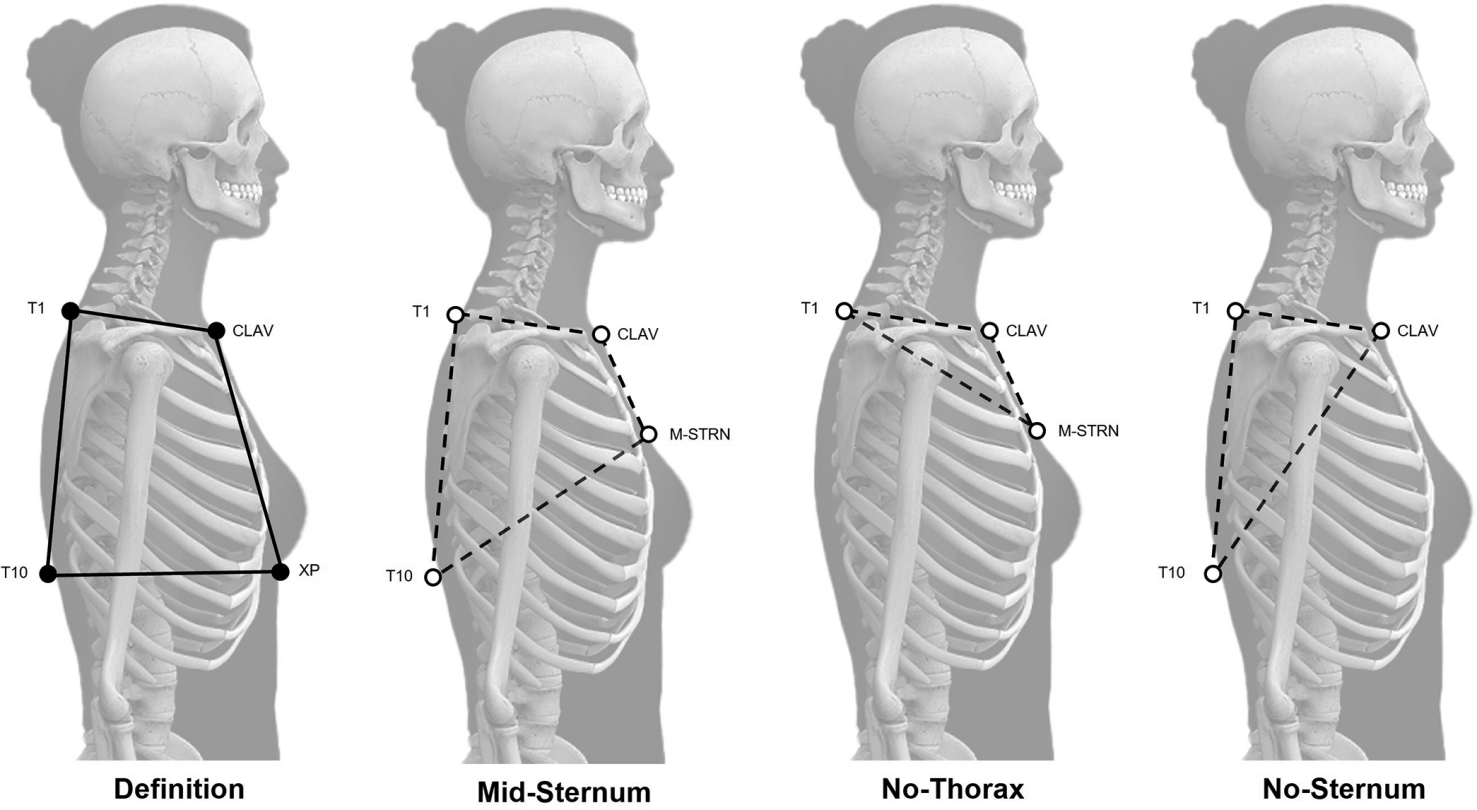
Tracking model variations. Definition Model (left; marker set for static trial) indicated with black circles and tracking marker set variations indicated with white circles for the Mid-Sternum (M-STRN), No-Thorax, and No-Sternum model variations.

The current study presents an evaluation specifically on marker set variations for tracking the trunk segment during dynamic trials. While the trunk segment was defined in the static trial using the recommended marker set [Definition Model: CLAV/XP/T1/T10; Figure 1; (21)], variations for tracking the trunk segment included the Mid-Sternum (M-STRN) Model (CLAV/M-STRN/T1/T10), No-Thorax Model (CLAV/M-STRN/T1), and No-Sternum Model (CLAV/T1/T10). These three variations were specifically chosen to avoid difficulties with placement of the sternum and/or thorax markers in female participants due to sports bra coverage and/or anatomy-related occlusion (i.e., unrelated to specific movements).

### Participants

A convenience sample of twenty, recreationally active, adolescents and young adults (9 males, ages 13–27) were recruited from the local community and tested in a motion analysis laboratory housed within a sports medicine treatment facility. To be eligible, participants could not have an orthopedic condition or report a recent injury (within the past six months) that would limit their ability to walk or jump. For testing, participants were asked to wear comfortable attire and their personal athletic footwear. Female participants were tested in a sports bra or tight-fitting tank top (based on preference), and male participants were tested without a shirt. Lastly, this study was approved by an Institutional Review Board, and all participants provided informed written consent prior to initiating testing procedures.

### Procedures

Participants were instrumented with retroreflective markers placed on the following landmarks: jugular notch (CLAV), mid-sternum (M-STRN), xiphoid process (XP), and the 1st and 10th thoracic spinous processes (T1 and T10, respectively; Figure 1). Placement of M-STRN was identified as the midpoint between the CLAV and XP. For female participants, the XP was placed on the band of the sports bra, and the M-STRN was placed immediately above the sports bra neckline on the skin. If female participants requested to be tested in a tank, the fabric was rolled up and secured in the back with tape such that the T10 marker was placed on the skin. Additional markers were placed on the lower body according to a modified Cleveland Clinic marker set (27) to aid task event identification described below. A 14-camera Vicon motion capture system (Vicon Motion Systems Ltd, Oxford, UK) sampling at 240 Hz was used to collect kinematic data while participants performed over-ground walking and a DVJ.

All participants began data collection by performing a single static trial standing in a T-pose. For the gait task, participants were instructed to walk at a self-selected speed along a 10-meter walkway. Subsequently, participants were asked to perform three DVJ trials. The DVJ required participants to stand on a 31 cm high plyometric box positioned one-half of the participant's height away from the landing target, which was designated by two large squares (8). Participants were instructed to jump horizontally off the box, land and immediately perform a maximum vertical jump, then land back in the landing target.

### Data processing and analysis

Trials were processed in Vicon Nexus (Vicon Motion Systems Ltd, Oxford, UK), and a Woltring filter was applied to marker trajectories with a predicted mean square error of 10 mm^2^ (28). The anatomical trunk segment was defined in the static trial using the recommended marker set: CLAV, XP, T1, and T10. Subsequently, the technical coordinate systems used for tracking the trunk in dynamic trials were the M-STRN, No-Thorax, and No-Sternum Models. These three tracking models used a variation of three or four markers. In the static trial, rotation matrices were computed to reposition the technical coordinate system and estimate the orientation of the anatomical coordinate system. Specifically, for four-marker models, midpoint locations between the trunk markers were calculated and saved as virtual markers. Then, vectors were computed using the virtual (midpoint) markers and cross products were performed to create the coordinate system axes. Alternatively, for the three-marker models, vectors were computed using the surface markers and then a similar process was followed to create the coordinate system axes.

For each trunk model variation, 3D trunk angles were computed using a custom 6-degree-of-freedom model. Gait cycle events were indicated at heel strike and toe-off, and two DVJ events were identified at initial contact with the floor and toe-off as the participant entered flight phase of the maximum vertical jump. For each participant, a single representative trial was selected per task for analysis. 3D measures of trunk motion were computed across the gait and DVJ cycles, including range-of-motion (ROM), maximum, minimum, and average trunk angles.

### Statistical analysis

For descriptive purposes, trunk tilt measures computed from gait and the DVJ were averaged across participants for each trunk model variation and for females and males separately. Sex differences in trunk kinematics across trunk model variations were not statistically evaluated in the current study given the limited sample size. The tracking models evaluated in comparison to the recommended Definition Model included: M-STRN Model, No-Thorax Model, and No-Sternum Model. The mean difference for each paired comparison was computed across trunk tilt measures by calculating the model-to-model difference for each participant and averaging the differences across the cohort. Given significant normality tests (i.e., Shapiro-Wilk), nonparametric analyses were used. Wilcoxon signed-rank tests were performed to determine significant differences between the definition model and each tracking model. Subsequently, intraclass correlation coefficients (ICC; 95% CI) of a two-way mixed-effects model were computed in SPSS (IBM Corp., Armonk, NY) per tracking model for sagittal plane kinematic variables (mean, maximum, minimum, and ROM of trunk tilt). Agreement indicated whether the kinematic measures from each tracking model variation were similar to the Definition Model which was also used to define the trunk in the static trial. ICC values were interpreted as poor (<0.50), moderate (0.50–0.75), good (0.75–0.90), or excellent (>0.90) (22, 29). Significance level (*α*) was set to 0.05.

## Results

**Figure 2:**
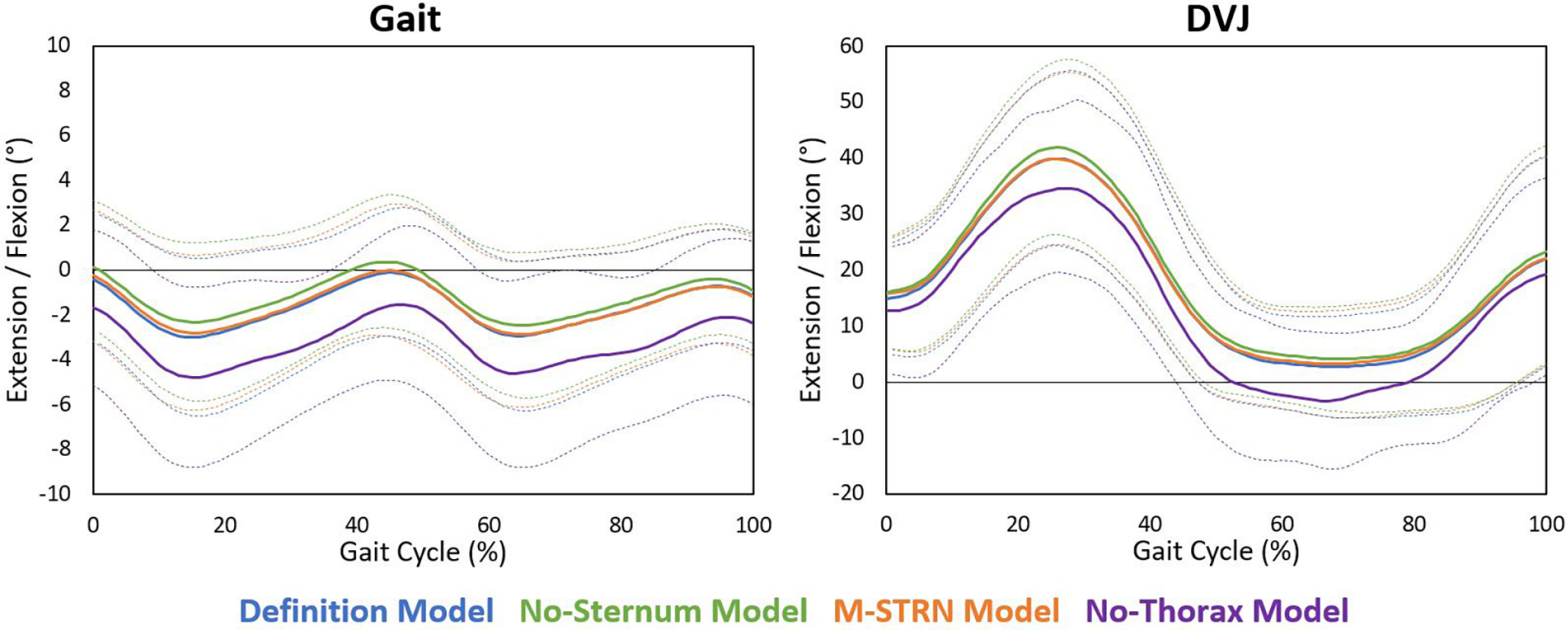
Average sagittal plane trunk angles across trunk model variations during gait and the DVJ. Each solid curve represents the average trunk tilt angle across participants for each model variation. Light dashed curves represent one standard deviation above and below the average curve for each model variation. Standard deviation curves were generated by computing the standard deviation of the trunk tilt angle across participants at each 1% of the gait cycle for each model variation.

**Figure 3:**
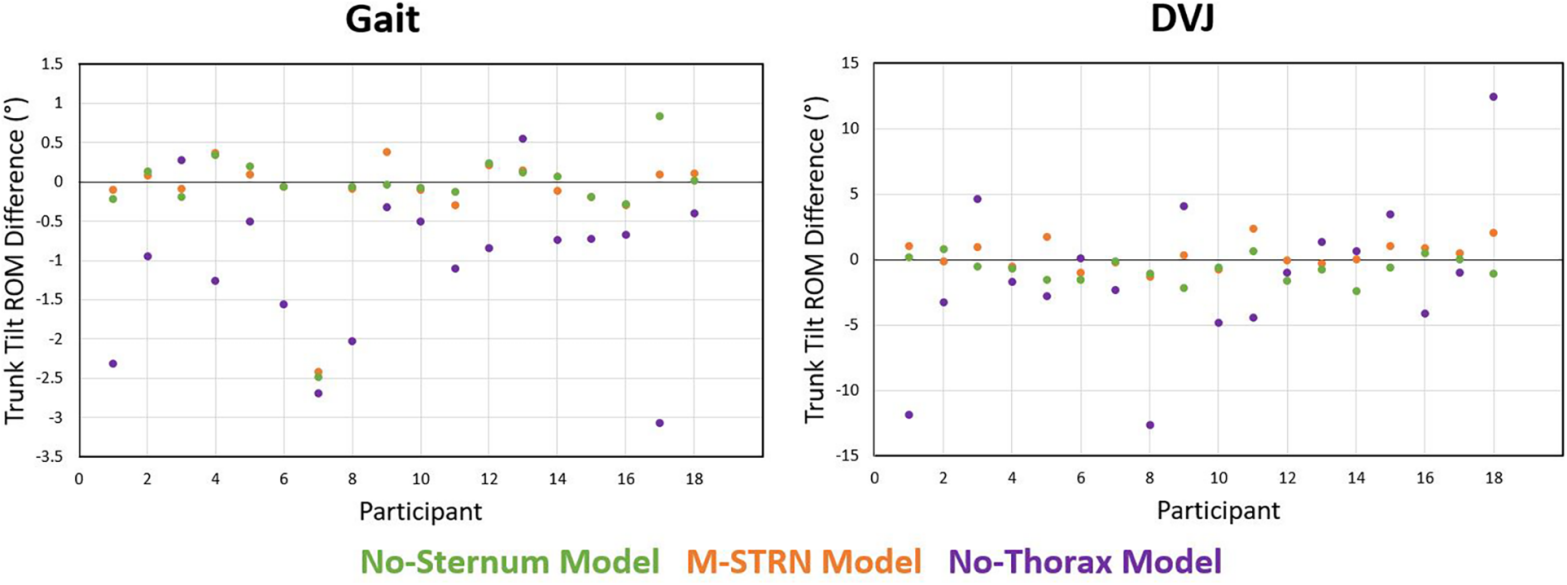
Differences in trunk tilt ROM between the Definition Model and each trunk model variation during gait and the DVJ.

Eighteen participants (7 males; age: 21.8, SD 4.1 years; height: 168.6, SD 10.4 cm; weight: 68.2, SD 15.3 kg) were included for analysis. Two participants (two males) of the twenty tested were excluded from the original sample due to marker dropout unrelated to trunk marker occlusions (e.g., poor calibration of the motion capture system resulted in flickering markers across the full marker set). During gait, the largest discrepancy between the Definition Model and tracking model variations was observed with the No-Thorax model as trunk tilt appeared considerably more extended (Figure 2), and significantly different trunk tilt measures were computed between models (Table 1, Figure 3). While the majority of differences were minimal (mean differences: 1.14°–2.27°), only moderate to good agreement was found (ICCs: 0.65–0.75; Table 2). Alternatively, the smallest differences were found with the M-STRN model (mean differences: 0.26°–0.37°) which also exhibited excellent agreement (ICCs: 0.94–0.99) across kinematic measures. Regarding sex differences, trunk tilt measures computed from the Definition Model differed between males and females by 0.37°–1.61° (Table 3). The M-STRN and No-Sternum models elicited similar results (mean sex differences: 0.35°–1.89° and 0.16°–1.78°, respectively) while the No-Thorax model resulted in a larger discrepancy between sexes (mean difference: 0.24°–2.58°). However, all sex differences in trunk tilt measures from gait were found to be relatively minimal.

**Table 1:** Mean differences in trunk tilt measures between the Definition Model and trunk model variations during gait and a DVJ.

Variable	Gait		Drop vertical jump	
	Mean diff. (°)	*p*-value	Mean diff. (°)	*p*-value
M-STRN Model				
Mean	0.26	0.349	0.94	0.327
Max	0.35	0.528	0.97	0.472
Min	0.37	0.744	1.00	0.102
ROM	0.29	0.811	0.84	0.248
No-Thorax Model				
Mean	1.88	**0**.**006**	5.44	**<0** **.** **001**
Max	1.61	0.078	4.73	**0**.**001**
Min	2.27	**0**.**003**	5.79	**0**.**001**
ROM	1.14	**0**.**001**	4.25	0.267
No-Sternum Model				
Mean	0.61	**0**.**006**	1.96	**<0** **.** **001**
Max	0.67	**0**.**008**	1.96	**<0** **.** **001**
Min	0.59	**0**.**020**	1.35	**0**.**001**
ROM	0.32	0.845	0.94	**0**.**011**

Significant differences (*p* < 0.05) noted in bold.

The Definition Model consists of CLAV, XP, T1, and T10. Alternatively, model variations include the M-STRN Model (CLAV/M-STRN/T1/T10), No-Thorax Model (CLAV/M-STRN/T1), and No-Sternum Model (CLAV/T1/T10). The maximum, minimum, and range-of-motion (ROM) measures of trunk tilt were extracted from a single gait cycle and from initial contact to take-off of the first landing during a single DVJ trial. Mean trunk tilt was also computed across the gait cycle and recorded at maximum descent of the first landing of the DVJ.

**Table 2:** Agreement between the Definition Model and trunk model variations based on trunk tilt measures during gait and a DVJ.

Variable	Gait		Drop vertical jump	
	ICC	95% CI	ICC	95% CI
M-STRN Model				
Mean	0.99	0.98–1.00	1.00	0.99–1.00
Max	0.98	0.96–0.99	1.00	0.99–1.00
Min	0.99	0.97–1.00	0.98	0.96–0.99
ROM	0.94	0.85–0.98	1.00	0.99–1.00
No-Thorax Model				
Mean	0.68	0.20–0.88	0.90	0.34–0.97
Max	0.75	0.44–0.90	0.94	0.52–0.98
Min	0.65	0.11–0.87	0.57	0.02–0.84
ROM	0.74	0.07–0.92	0.89	0.73–0.96
No-Sternum Model				
Mean	0.97	0.83–0.99	0.99	0.46–1.00
Max	0.96	0.82–0.99	0.99	0.44–1.00
Min	0.97	0.89–0.99	0.97	0.64–0.99
ROM	0.93	0.83–0.97	1.00	0.97–1.00

The Definition Model consists of CLAV, XP, T1, and T10. Alternatively, model variations include the M-STRN Model (CLAV/M-STRN/T1/T10), No-Thorax Model (CLAV/M-STRN/T1), and No-Sternum Model (CLAV/T1/T10). ICC values greater than 0.90 (white), between 0.90 and 0.75 (light grey), between 0.75 and 0.50 (medium grey), and less than 0.50 (dark grey) represent excellent, good, moderate, and poor agreement, respectively. The maximum, minimum, and range-of-motion (ROM) measures of trunk tilt were extracted from a single gait cycle and from initial contact to take-off of the first landing during a single DVJ trial. Mean trunk tilt was also computed across the gait cycle and recorded at maximum descent of the first landing of the DVJ.

**Table 3:** Average trunk tilt measures (degrees) across trunk models during gait and a DVJ.

Variable	Gait			Drop Vertical Jump		
	ALL	Female	Male	ALL	Female	Male
Definition Model						
Mean	−1.68 ± 2.80	−1.83 ± 2.34	−1.44 ± 3.61	37.33 ± 15.57	29.34 ± 13.13	49.88 ± 9.93
Max	0.51 ± 2.88	0.66 ± 2.27	0.29 ± 3.85	41.95 ± 16.01	34.43 ± 13.64	53.76 ± 12.24
Min	−3.83 ± 3.16	−4.31 ± 2.78	−3.07 ± 3.78	−1.99 ± 6.55	−3.87 ± 7.39	0.96 ± 3.74
ROM	4.34 ± 1.79	4.97 ± 2.05	3.36 ± 0.47	43.94 ± 14.07	38.30 ± 11.42	52.81 ± 13.91
M-STRN Model						
Mean	−1.60 ± 2.77	−1.74 ± 2.28	−1.39 ± 3.61	37.45 ± 15.49	29.12 ± 12.48	50.53 ± 9.67
Max	0.65 ± 2.95	0.86 ± 2.40	0.32 ± 3.85	42.04 ± 15.79	34.13 ± 12.81	54.45 ± 11.76
Min	−3.82 ± 3.04	−4.34 ± 2.51	−2.99 ± 3.78	−1.51 ± 6.70	−4.13 ± 6.94	2.62 ± 3.78
ROM	4.47 ± 1.69	5.20 ± 1.80	3.31 ± 0.35	43.54 ± 13.42	38.27 ± 11.18	51.83 ± 13.05
No-Thorax Model						
Mean	−3.29 ± 3.37	−3.81 ± 2.85	−2.48 ± 4.18	32.16 ± 14.99	24.42 ± 12.04	44.32 ± 10.62
Max	−0.49 ± 3.49	−0.58 ± 3.06	−0.35 ± 4.34	37.87 ± 16.12	30.47 ± 13.23	49.50 ± 13.67
Min	−5.88 ± 3.71	−6.88 ± 2.97	−4.30 ± 4.43	−8.91 ± 9.21	−12.51 ± 9.53	−3.25 ± 5.28
ROM	5.39 ± 1.85	6.30 ± 1.79	3.96 ± 0.67	46.78 ± 14.33	42.98 ± 11.98	52.75 ± 16.57
No-Sternum Model						
Mean	−1.21 ± 2.79	−1.27 ± 2.52	−1.11 ± 3.38	39.29 ± 15.77	31.09 ± 13.37	52.18 ± 9.47
Max	1.03 ± 2.96	1.30 ± 2.55	0.59 ± 3.69	43.93 ± 16.02	36.26 ± 13.65	55.99 ± 11.78
Min	−3.41 ± 3.08	−3.83 ± 2.81	−2.76 ± 3.59	−0.70 ± 6.85	−2.81 ± 7.57	2.62 ± 4.07
ROM	4.44 ± 1.68	5.13 ± 1.82	3.35 ± 0.42	44.63 ± 13.81	39.06 ± 11.42	53.37 ± 13.31

3D trunk angles reported in degrees. Values represent the average angle across all participants as well as female and male participants separately for each trunk model. Negative values indicate trunk extension. The Definition Model consists of CLAV, XP, T1, and T10. Alternatively, model variations include the M-STRN Model (CLAV/M-STRN/T1/T10), No-Thorax Model (CLAV/M-STRN/T1), and No-Sternum Model (CLAV/T1/T10). The maximum, minimum, and range-of-motion (ROM) measures of trunk tilt were extracted from a single gait cycle and from initial contact to take-off of the first landing during a single DVJ trial. Mean trunk tilt was also computed across the gait cycle and recorded at maximum descent of the first landing of the DVJ.

For the DVJ, kinematic differences computed between the Definition and No-Thorax models ranged from 4.25° to 5.79°. However, only two of the four measures used to evaluate kinematic agreement between models indicated below excellent agreement. Minimum trunk tilt exhibited moderate agreement (ICC: 0.57) and trunk tilt ROM exhibited good agreement (ICC: 0.89) between models. Alternatively, kinematic discrepancies for the M-STRN and No-Sternum models were minimal with mean differences ranging from 0.84° to 1.00° and 0.94° to 1.96°, respectively. Similarly, both models exhibited excellent agreement with the Definition model (M-STRN ICCs: 0.98–1.00; No-Sternum ICCs: 0.97–1.00). Between males and females, trunk tilt measures across all models were considerably greater in males likely due to differences in movement strategy (mean difference in maximum trunk tilt is approximately 20°). However, this discrepancy between sexes was consistent across models with a notable exception. Specifically, trunk tilt ROM across the DVJ cycle (initial contact to take-off of first landing) in female participants was greater with the No-Thorax model relative to other models, and thus, the difference between males and females was reduced. The Definition, M-STRN, and No-Sternum models exhibited differences between sexes of 14.51°, 13.57°, and 14.31°, respectively. Alternatively, the No-Thorax model only indicated a 9.77° difference in trunk tilt ROM between males and females (∼4° less).

## Discussion

The purpose of this study was to determine whether specific marker placement adjustments were consistent in tracking the trunk segment during dynamic trials compared to the definition model. During dynamic testing, certain marker locations (i.e., XP and T10) may become occluded or shift during dynamic trials due to the type of clothing worn or body size (e.g., sports bra or tank top coverage, adipose tissue). Therefore, the current study evaluated the potential to adjust marker placement or remove problematic markers during dynamic trials. Briefly, the M-STRN model, in which the XP marker was adjusted to the M-STRN placement, exhibited the least discrepancies and excellent agreement with the Definition Model during both gait and the DVJ. Alternatively, the model in which the thorax marker was removed (No-Thorax model) performed the worst exhibiting relatively greater kinematic differences during the DVJ and moderate to excellent agreement across both tasks.

Although the trunk segment is modeled as a rigid body, intra-segmental motion is inherently present. However, this is captured at varying levels across the tracking models and greatly dependent on the individual markers used to border the trunk segment. While the trunk was defined in the static trial using the T10 marker, it was hypothesized that the thoracic marker may not be necessary for tracking during dynamic trials. If so, removing the T10 marker after the static trial would be convenient and more comfortable for participants who prefer testing in a tank. Orantes-Gonzalez et al. compared a custom trunk model that consisted of only anterior trunk markers (i.e., sternum, acromion), in addition to pelvis markers, to the standard ISB model (22). While frontal and transverse plane kinematic differences were negligible with good to excellent agreement (ICCs: 0.88–0.97), agreement in trunk flexion (ICC: 0.64) and extension (ICC: 0.55) was only moderate.

Similarly, in the current study, the No-Thorax model failed to capture the full range of the thoracic trunk, as seen in the reduced trunk tilt ROM during the DVJ. Based on the presented findings, a tracking model consisting of only the most superior marker placements (CLAV/M-STRN/T1) is insufficient. A more inferior placement on the posterior trunk, specifically the T10 placement, is necessary to accurately track the trunk segment. The authors also recommend maintaining a four-marker model, including a sternum marker, as the No-Sternum Model (CLAV/T1/T10) did not perform as well as the M-STRN Model (CLAV/M-STRN/T1/T10) compared to the Definition Model.

While the current study only considered marker placements along the midline and thus primarily evaluated sagittal plane trunk kinematics, it is important to note that alternative trunk marker sets reported in the literature have included non-axial marker placements as well. For example, Leardini et al. compared eight trunk marker sets commonly used to model trunk kinematics during activities of daily living that varied from a total of two to eight markers and included placements along the midline, both anteriorly and posteriorly, as well as on the acromion bilaterally and lower lumbar spine (30). They reported that range-of-motion differed across trunk model variations and discrepancies varied by task. During a sit-to-stand task, range-of-motion in the sagittal plane differed up to 16° across models. Additionally, while midline models that were primarily in the thorax region were found to overlook motion of the shoulders and/or subtle movements outside of the sagittal plane, full trunk models (including the acromion and lower lumbar markers) commonly failed to represent thorax motion and were sensitive to movement by the upper extremities (30). Their findings emphasize the need to consider marker locations and reference frame definitions based on the tasks evaluated and the ultimate clinical interpretation. Future work should evaluate the effects of incorporating non-midline or lateral trunk markers, such as mid-clavicle markers (31), on frontal and transverse plane kinematics specifically for sports testing.

A few limitations of the current study should be noted. Although discrepancies between males and females were presented across model variations for both gait and the DVJ, sex differences were not statistically evaluated given the limited sample size of the current study. Overall, relatively minor differences across trunk tilt measures were observed between males and females, with the No-Thorax model eliciting the most inconsistencies. As the recommended model for tracking the trunk segment during dynamic trials includes the M-STRN marker, occlusion concerns with the XP marker placement will likely be resolved. However, the sensitivity of the recommended trunk model to slight deviations in placement of the M-STRN marker should be evaluated in future work, especially in female participants and across a variety of tasks. Future studies should also recruit larger sample sizes with a diverse distribution of participants across sex, age, activity level, and body type or size. Larger sample sizes will ensure adequately powered results, and evaluating trunk model sensitivity to different body types and sizes will validate the generalizability of the findings. Furthermore, trunk segment angles were computed using a single method (e.g., midpoint computations to define axes, transverse axis used as permanent axis, transverse-coronal-sagittal rotation sequence, global segment definition). It is important to note that in addition to marker placement differences, methods for computing trunk segment angles also differ across motion capture labs and therefore should be evaluated in future work.

## Conclusion

Based on the findings of the current study, the XP marker may be slightly adjusted prior to the collection of dynamic trials to reduce marker occlusion or drop out on the sternum. Specifically, whether the XP marker or a dipstick is used to define the trunk segment during the static trial, the M-STRN marker location can be used for tracking the trunk during dynamic trails. The M-STRN should be placed on the skin at the midpoint between the CLAV and XP locations. If placement on the skin is not available, it may be moved up immediately above the sports bra or tank neckline. The remaining three markers of the Definition Model (CLAV, T1, and T10) are all required to accurately model the trunk. If the sports bra band interferes with placement of the T10, a superior adjustment up to T8 is appropriate, but marker placement should not exceed superiorly past T8 or fall below T10. Finally, the authors have provided trunk and lower extremity marker placement recommendations as well as details for how to model trunk and lower extremity kinematics specifically for sports testing in an online manual [Pediatric Research in Sports Medicine (PRiSM), Motion Analysis Research Interest Group (RIG), Sports Protocol: Knee Emphasis Standard Operating Procedures; (21)].

## Data Availability

The raw data supporting the conclusions of this article will be made available by the authors, without undue reservation.
